# Cardiothoracic Surgery in the Caribbean

**DOI:** 10.21470/1678-9741-2020-0377

**Published:** 2021

**Authors:** Eric E. Vinck, Tjark Ebels, Romain Hittinger, Tim F. Peterson

**Affiliations:** 1 Department of Cardiovascular Surgery, Clínica Cardio VID, Medellín, Colombia.; 2 Department of Cardiothoracic Surgery, Groningen UMC, Groningen, Netherlands.; 3 Department of Cardiovascular Surgery, Martinique University Hospital, Fort-de-France, Martinique.; 4 Department of Surgery, Dr. Horacio Oduber Hospital, Oranjestad, Aruba.

**Keywords:** Caribbean Region, Thoracic Surgery, PubMed, MEDLINE, Local Government, Surgeons, Cardiac Surgery Procedures

## Abstract

**Introduction:**

Despite being one of the main vacation destinations in the world, health care in the Caribbean faces many difficulties. The challenges involved in these islands’ medical care range from low-resource institutions to lack of specialized care. In the field of thoracic and cardiac surgery, many limitations exist, and these include the lack of access to cardiac surgery for many small islands and little governmental funding for minimally invasive approaches in thoracic surgery.

**Methods:**

Literature review was done using PubMed/MEDLINE and Google Scholar databases to identify articles describing the characteristics of thoracic and cardiac surgery departments on Caribbean islands. Articles on the history, current states of practice, and advances in cardiothoracic surgery in the Caribbean were reviewed.

**Results:**

Regardless of the middle to high-income profile of the Caribbean, there are significant differences in the speed of technological growth in cardiothoracic surgery from island to island, as well as disparities between the quality of care and resources. Many islands struggle to advance the field of cardiothoracic surgery both through lack of local cardiac surgery centers and limited financial funding for minimally invasive thoracic surgery.

**Conclusions:**

Cardiac and thoracic surgery in the Caribbean depend not only on the support from local government policies and proper distribution of healthcare budgets, but efforts by the surgeons themselves to change and improve institutional cultures. Although resource availability still remains a challenge, the Caribbean remains an important region that deserves special attention with regard to the unmet needs for long-term sustainability of chest surgery.

**Table:** 

Abbreviations, acronyms & symbols	
CABG	= Coronary artery bypass grafting
CCS	= Caribbean Cardiac Society
CT	= Cardiothoracic
ECMO	= Extracorporeal membrane oxygenation
EuroSCORE	= European System for Cardiac Operative Risk Evaluation
GNI	= Gross national income
HI	= High income
LI	= Low income
LVAD	= Left ventricular assist device
MI	= Middle income
MICS	= Minimally invasive cardiac surgery
VATS	= Video-assisted thoracoscopic surgery

## INTRODUCTION

Despite being one of the main vacation destinations in the world, health care in the Caribbean faces many difficulties. The challenges involved in these islands’ medical care range from low-resource institutions to lack of specialized care. In the field of thoracic and cardiac surgery, many limitations exist, and these include the lack of access to cardiac surgery for many small islands, little governmental funding, and limited health budgets for minimally invasive approaches in thoracic surgery^[1]^. The Caribbean is a region consisting of 44 million inhabitants. Most of these islands are middle to high-income primarily, with a very small percentage of them being low-income islands. Despite the general “high-income” region profile of Caribbean islands, many difficulties surface with regard to both access to and quality of specialized care, particularly regarding cardiac and thoracic surgery^[1]^. Despite a great disparity between low and high-income islands, both cardiac and thoracic surgery have existed in the Caribbean for over 50 years. The availability of cardiac and thoracic surgery is different from island to island. Most islands with populations over 500,000 perform video-assisted thoracoscopic surgery (VATS) and have local cardiac surgical centers^[1]^. These include Puerto Rico, Cuba, Jamaica, and Trinidad and Tobago. In contrast, smaller islands are less likely to have cardiac and thoracoscopic surgery at their hospitals. Exceptions include the Cayman Islands (population of 65,000), which have been performing cardiac surgery since 2014, and Aruba (population of 105,000), which performs VATS^[1]^. These islands are supported extensively by their local governments, which facilitates technological developments in cardiothoracic (CT) surgery. On the other hand, Haiti, a low-income island with 11 million people, has not been able to establish sustainable cardiac surgery, and thus relies on patient transfers and visiting cardiac surgery teams to meet the demands of CT surgical disease^[1]^. According to the latest classifications of the World Bank, high-income countries are defined as earning > US$12,370 gross national income (GNI) per capita, middle-income countries as between US$3,996 and US$12,370 GNI per capita, and low-income countries as < US$3,995 GNI per capita^[2]^. The two main factors determining which islands have advanced CT care are island population and state of income^[1]^. This paper overviews the current situation of CT surgery in the Caribbean and highlights the importance of both domestic and overseas support.

## METHODS

Literature review was done using PubMed/MEDLINE and Google Scholar databases to identify articles describing different characteristics of thoracic and cardiac surgery practice on Caribbean islands. Articles on the history, current states, and advances in CT surgery in the Caribbean were reviewed. Chosen manuscripts included those describing cardiac and thoracic surgery volumes and the individual aspects on different islands in the Caribbean including the challenges and difficulties of performing cardiac and thoracic surgery concerning resource availability and the remote distances from specialized centers. Articles were collected and a general description of the practice of CT surgery is described focusing on the need for technological advances in Caribbean chest surgery.

## RESULTS

### Cardiac Surgery on Caribbean Islands

The earliest cardiac surgery centers known in the Caribbean date back to 1951 in Havana, Cuba (*Instituto de Cirugía Cardiovascular y Torácica*)^[3]^. In Jamaica, cardiac surgery has been reported since 1968, and in Puerto Rico since the late 1980s^[4]^. In 1988, Martinique opened its cardiac surgery department in Fort-de-France. In 1992, the *Centro Cardiovascular de Puerto Rico y del Caribe* was established and started to perform the first cardiovascular surgeries under an official cardiovascular surgery department, and in 1999 they performed the island’s first heart transplant. In 1993, Trinidad and Tobago opened the island’s first heart surgery center^[5]^. In the Bahamas, the first valve replacement surgeries were performed in 1994, and the first coronary artery bypass grafting (CABG) took place in 1996. Also, in 1994, Barbados opened the island’s first cardiac surgery center. By 1994, Jamaica performed its first CABG and began implementing CT surgery residency into its curriculum. Today, there are only three Caribbean islands with CT residency programs: Cuba, Jamaica, and Martinique. Other Caribbean practicing cardiac surgeons train in the United States of America, Europe, and in Latin American countries^[6,1]^.

The first cases of minimally invasive cardiac surgery (MICS) reported in the Caribbean were in Cuba, in 2011, and nowadays Jamaica, Martinique, and the Cayman Islands are also performing MICS. In 2014, the Cayman Islands would go on to perform the first left ventricular assist device (LVAD) placement in the Caribbean. In 2015, Martinique performed the island’s first video-assisted mitral valve surgeries as well as Perceval aortic valve replacement surgeries through a mini-thoracotomy. Jamaican cardiac surgeons performed the island’s first MICS in 2016; the first cases were aortic valve replacements using a mini-sternotomy. In comparison to other developing mainland countries, Caribbean islands have not lagged behind significantly. Many of the surrounding mainland countries have also been in development, for instance, Colombia began performing minimally invasive and thoracoscopic cardiac surgeries during the mid-1990s and those became routine in 2010^[7]^. Table 1 highlights important events in the Caribbean cardiac surgery timeline.

**Table 1:** Caribbean cardiac and thoracic surgery highlights.

Island	Highlights
Jamaica	First cardiac surgeries (1968)
	Cardiothoracic surgery training program (1990s)
	First minimally invasive cardiac surgery (2016)
Trinidad and Tobago	First cardiac surgery (1993)
Barbados	First cardiac surgery (1994)
Bahamas	First cardiac surgery (1994)
Cayman Islands	First Caribbean LVAD placement (2014)
	Performs MICS and VATS
	ECMO air-bridging
Puerto Rico	First cardiac surgery (1992)
	First heart transplant (1999)
Cuba	First cardiac surgeries in the Caribbean (1951)
	Established cardiac surgery training programs
	First minimally invasive cardiac surgery (2011)
Aruba	First VATS lobectomy in the Dutch Caribbean
Martinique	Only cardiac surgery institution in the French Caribbean (since 1988)
	Performs MICS (2015) and VATS
	Has a Cardiac surgery residency program
	ECMO air-circulatory support program
	First studies on the EuroSCORE
	Robotic cardiac surgery (2021)

ECMO=extracorporeal membrane oxygenation; EuroSCORE=European System for Cardiac Operative Risk Evaluation; LVAD=left ventricular assist device; MICS=minimally invasive cardiac surgery; VATS=video-assisted thoracoscopic surgery

With regard to the access to cardiac surgical care, larger Caribbean islands with populations over 500,000, such as Jamaica, Trinidad and Tobago, Cuba, and Puerto Rico, have local cardiac surgery. Therefore, they do not rely on sustained foreign assistance. Many of the smaller Caribbean islands, however, rely on cardiac surgery air-bridging for overseas care or visiting cardiac surgery teams. These include the Dutch Caribbean islands, Turks and Caicos, and the Virgin Islands, among others^[1]^. In the Caribbean, the 44 million inhabitants receive cardiac surgery through three different methods: 1) local cardiac centers, 2) visiting cardiac teams, and 3) air-bridging. Approximately 30.5 million of the Caribbean population have direct access to cardiac surgery through the 30 local cardiac surgery centers. Another 12 million are almost completely dependent on visiting cardiac surgical teams, and the remaining 1.5 million are dependent on air-bridging^[1]^. An estimated volume of roughly 1,500 patients per year require air-bridging in the Caribbean for heart surgery. Therefore, in the Caribbean, there is sufficient patient volume for an additional five cardiac surgery centers^[1]^.

**Fig. 1:**
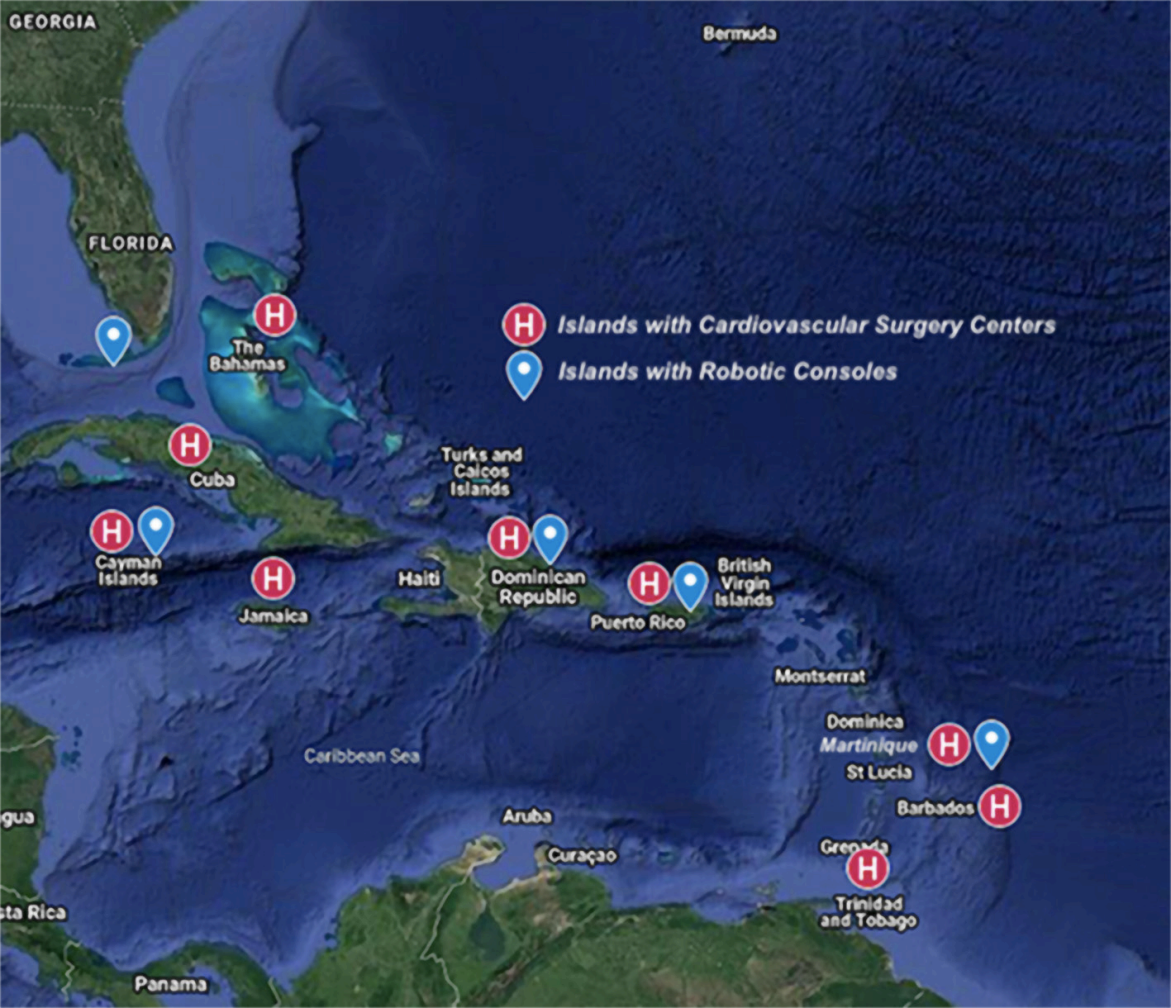
Distribution of Caribbean islands with cardiovascular surgery centers and robotic consoles.

In special cases, the Dutch islands air-bridge patients to Amsterdam, and Martinique to Paris, on extracorporeal membrane oxygenation (ECMO) systems through Air France flights. Figure 1 displays the Caribbean islands with cardiovascular surgery centers.

As more attention is being drawn to the global unmet needs of cardiac surgery disease burden, especially in low-income countries as well as the Caribbean region, the possibility of establishing local cardiac surgery centers on smaller islands is evolving^[1]^. Traveling cardiac surgery teams include organizations such as *Heart to Heart Mission, Haiti Heart Alliance, CardioStart International, Caribbean Heart Menders*, and *Hearts for Haiti*, among others^[1]^. However, although traveling cardiac surgeons offer a decent solution, only a small number of patients benefit from this system and they serve only as a temporary, unsustainable solution. In addition, visiting cardiovascular surgeons have to adapt to commonly less than optimal working conditions with limited resources, as well as inexperienced surgical personnel^[1]^. Table 2 lists the different forms of cardiac surgery accesses on Caribbean islands according to GNI per capita.

**Table 2:** Caribbean islands with cardiac surgery centers, visiting teams, and air-bridge dependencies^[^1^]^.

Local cardiac surgery	GNI/Capita	Air-bridge dependent	GNI/Capita	Visiting team dependent	GNI/Capita
Jamaica	9.520 (MI)	St. Barths, St. Martin, and French West Indies	26.855 (HI)	Haiti	1.810 (LI)
Trinidad and Tobago	26.050 (HI)	British Virgin Islands	34.246 (HI)		
Puerto Rico	23.620 (HI)	United States Virgin Islands	35.938 (HI)		
Cuba	7.480 (MI)	Bermuda	59.483 (HI)		
Barbados	14.140 (HI)	Turks and Caicos	27.075 (HI)		
Bahamas	34.370 (HI)	Aruba, Curaçao, and Caribbean Netherlands	33.966 (HI)		
Cayman Islands	41.640 (HI)	San Andrés, Providence, and Sta. Catalina	1.072 (LI)		
Dominican Republic	17.330 (HI)	Saint Lucia & Grenada	13.960 (HI) & 17.234 (HI)		
Martinique	27.688 (HI)	Antigua & Barbuda	21.630 (HI)		
		Haiti	1.810 (LI)		
		St. Vincent & The Grenadines	12.770 (HI)		
**30.5 million**		**1.5 million**		**12 million**	
Other: Florida Keys (ground transport to mainland Florida)					35.516 (HI)

GNI=gross national income; HI=high income; LI=low income; MI=middle income

The Caribbean has very few societies organizing surgical and cardiovascular events including regional practice and developments. The two most important societies are the Caribbean Society of Endoscopic Surgeons, born in 2013 on the island of Curaçao, and the Caribbean Cardiac Society (CCS), founded in 1988 in Jamaica^[8,9]^. The CCS holds annual congresses in different islands throughout the Caribbean and works in association with the European Heart Society. In Martinique and Aruba, videoconferences are used to maintain permanent communications with the French and Dutch CT societies.

### Thoracic Surgery in the Caribbean

The four main reasons for the delay in developing VATS in the Caribbean have been: 1) resource availability, 2) poorly trained operating room staff, 3) low case volume, and 4) institutional culture^[9-12]^. For the English-speaking Caribbean, the three islands with the most experience in VATS have been Jamaica, Barbados, and Trinidad and Tobago. With a mortality rate approaching zero and an 8.6% conversion rate to open surgery, these islands offer statistics paralleling those in developed countries at high-volume centers^[9-12]^. In surrounding mainland developing countries, VATS has also lagged behind high-income countries. Colombia, for example, began performing formal VATS resections in the early 2000s^[13]^.

In Barbados, VATS is performed at the Queen Elizabeth Hospital, the only hospital on the island with a surgical intensive care unit. Although VATS in Barbados began in 1996, these were mostly pleural and diagnostic. Despite these endeavors to enhance thoracic surgery, because of the lack of endoscopic staplers, no lobar pulmonary resections have been performed up to 2019^[9-12]^. In 2016, uniportal VATS began on the island. Because this hospital is governmentally funded, limited budgets do not allow for quick advances, as a matter of fact, thoracoscopy is performed using laparoscopic equipment and even pleurodesis is done using mechanical abrasion because of the lack of chemical solutions for pleurodesis^[9-12]^. In a retrospective analysis, the experience with 50 patients over the period of May 1996 to February 2003 was described^[9-12]^. This study looked at various factors and outcomes in an attempt to ascertain the viability of VATS procedure on the island. There were 24 males and 26 females included in the study. VATS was used for diagnosis in 27 cases (54%), therapeutic indication in 17 cases (34%), and as both a diagnostic and therapeutic modality in six cases (12%). In 92% of cases, the operations were completed thoracoscopically with a conversion rate of 8%. The morbidity and mortality rates were 18% and 2%, respectively^[9-12]^.

In Jamaica, thoracic and cardiac surgery are entwined into one subspecialty as CT surgery, such as in the Netherlands. In Jamaica, only 13% of thoracic procedures are performed using a VATS approach at the University Hospital of the West Indies^[9-12]^. Jamaican thoracic surgery faces the same challenges as Barbados with regard to equipment availability, to the extent that some patients have to purchase some disposable material needed themselves^[9-12]^. For now, thoracic surgery and VATS in Jamaica are still looking forward to expand to anatomical resections using VATS.

In Trinidad and Tobago, thoracic surgery evolved into a separate specialty in 1962 because of the need for a center with experience in tuberculosis. By 1994, most thoracic surgical procedures were moved to the Eric Williams Medical Sciences Complex (or EWMSC) along with the development of cardiac surgery on the island^[9-12]^. Although thoracoscopic procedures began in the 1960s, these were mostly diagnostic (pleuroscopy). By 2008, formal VATS procedures began with the acquisition of endoscopic staplers, however formal lobar pulmonary resections are still being performed through thoracotomy^[9-12]^. Other islands, like Cuba, have reports of video-assisted thoracic surgery since 2003.

In Curaçao, general thoracic surgery began in the 1980s through open incisions. In Aruba, a high-income island, thoracoscopic surgery has been performed since the late 1990s. Procedures included segmentectomies, bullectomies, wedge resections, and pleurodesis. Since the Dutch islands do not have CT surgeons, these general thoracic procedures are performed by general surgeons with training in thoracic surgery. Although acquiring endoscopic staplers and state-of-the-art equipment has not been a problem, formal VATS lobectomies did not begin until the final half of 2019, guided by visiting Dutch surgeons. Less than 10 Caribbean islands are currently performing formal lobar VATS resections. Table 3 lists the Caribbean islands performing minimally invasive thoracic and cardiac surgery.

**Table 3:** Caribbean islands performing minimally invasive cardiac and thoracic surgery.

Island	VATS	MICS	Islands with Robotic Consoles
Jamaica	+	+	-
Dominican Republic	+	+	+
Aruba (Dutch Antilles)	+	-	-
Puerto Rico	+	-	+
Trinidad and Tobago	+	-	-
Cuba	+	+	-
Florida Keys	-	-	+
Barbados	+	-	-
Bahamas	+	-	-
Cayman Islands	+	+	+
Martinique	+	+	+

MICS=minimally invasive cardiac surgery; VATS=video-assisted thoracic surgery

## DISCUSSION

### Should Caribbean Cardiothoracic Surgeons be Trained Differently?

Throughout the years, CT residency training has had different curricular structures and characteristics depending on the country’s epidemiological and demographic requirements^[7,13-15]^. Many Latin American programs have cardiac surgery training as a separate subspecialty from general thoracic surgery. Brazil and Colombia, for example, have cardiac and thoracic surgery as separate specialties^[7]^. In addition, some newer programs have integrated tracks straight from medical school without a prior general surgery training requirement^[7,13-15]^. In the Netherlands, CT surgery training is done straight from medical school as an integrated track combining both thoracic and cardiac surgery^[7,16]^. In the United States of America, two main possibilities exist, an integrated six-year CT training combining both thoracic and cardiac surgery and a three to four-year fellowship training following general surgery^[14,7]^. Regardless of the training structures, most surgeons streamline their practice depending on areas of interest and institutional, geographical, or population needs. Centers having an adequate number of surgeons can afford to have surgeons focusing their practice on areas such as robotics, transplant, mitral valve, aortic, lung resection, minimally invasive, and CABG, among others, without disrupting the balance of surgeon-to-patient ratio or cardiopulmonary surgical disease burden.

In the Caribbean however, special challenges exist regarding the “surgeon profile”, and there is generally little room for highly streamlined surgeons. Most surgeons working on islands are required to attend to a broader variety of pathologies of their specialty^[1,7]^. General surgeons, for example, are required to care for all scopes of general surgery such as colorectal surgery, vascular surgery, trauma/acute care surgery, and hepato-pancreato-biliary surgery. On some islands, general surgeons perform general thoracic procedures, such as open lobectomies, video-assisted thoracoscopic pleurodesis, sub-lobar resections, as well as decortication and pleurectomies.

Cardiac and thoracic surgeons practicing in the Caribbean receive training in the United States of America, Europe, Latin America, as well as in other Caribbean islands (Jamaica, Cuba, and Martinique)^[1,7,13,15]^. Regardless of their residency training, Caribbean CT surgeons are required to perform all kinds of cardiac surgeries including valve, coronary artery bypass, and aortic, in addition to general thoracic and pulmonary surgeries. Hospitals in the Caribbean are mostly government funded, thus surgeons are hired according to their capability of covering broader areas of thoracic pathologies, since institutions cannot afford to hire more surgeons based on cost-effectiveness^[1]^. The Caribbean CT surgeon, as a result, needs to be an “all around” surgeon to meet the demands of his/her island and be capable of facing many unfamiliar pathologies while adapting to limited resources. Consequently, cardiac and thoracic surgeons practicing on Caribbean islands have to constantly be aware of the need for external support and air-bridging whenever disease complexity surpasses local capacity for quality of care^[1]^. Also, if CT surgeons in training are planning to practice in the Caribbean, perhaps a broader oriented training curriculum is required instead of a streamlined training profile as seen in many developed mainland countries.

### The Future of Cardiothoracic Surgery in the Caribbean

### Robotics, ECMO, and Enhanced Recovery after Cardiac Surgery: are We Ready?

With regard to minimally invasive CT surgery, the Caribbean lags some 15 years behind developed countries. Despite the less than ideal governmental funding, health budgets, and support, technological advances in this region of the world has managed to maintain certain developments. In 2014, the Dominican Republic launched the first robotic surgical center in the Spanish-speaking Caribbean, second only Puerto Rico^[17,18]^. Although many areas of surgery are currently being treated using robotic consoles, robotic CT surgery in the Caribbean is still non-existent. According to the CCS registries, no Caribbean island is currently performing robotic cardiac procedures. By 2019, there were two consoles registered in Puerto Rico and another two in the Dominican Republic. The Florida Keys also have a robotic console, although some consider it to be part of mainland Florida. The Cayman Islands recently acquired a console; however, these institutions are still limited to non-cardiac robotic procedures^[17]^.

By 2017, there were 4,271 da Vinci robotic consoles worldwide with 2,770 of these being in the United States of America. In nearby developing countries, robotic CT surgery has only recently began developing^[17,18]^. Brazil began performing robot-assisted cardiac surgery in 2010, and Colombia in 2017^[7,18]^. Robot-assisted thoracic surgery began in Colombia and Brazil in 2012^[7,18]^. Robotic consoles are not cheap; depending on the model, a da Vinci robotic console may cost up to US$1.9 million and instrument parts can cost up to US$3,200 - needing replacement after 10 uses. In addition, surgical training, equipment maintenance, and repair, all add to the expenses of robotic surgery^[17]^. It has also been estimated that 150 surgeries per robot per year are required to maintain cost-effectiveness and profitability^[17]^. Although many of Intuitive Surgical’s patents are expiring, opening the market for competitors and thus more affordable consoles, the benefits of robotic cardiac and thoracic surgery over traditional minimally invasive CT surgery is still controversial. As a result, robotic CT surgery is still finding its place even in developed countries. Up and coming brands include Cambridge Medical Robotics, a British company developing consoles for thoracic surgery, Medical Microinstruments, an Italian company, Medtronic, Google, and Johnson & Johnson - all seeking to develop their robotic consoles. As these brands enter the market, the costs of robotic surgery will eventually decrease, allowing more and more lower income countries to provide affordable robotic surgery to their patients^[17]^. Figure 1 displays the Caribbean islands with robotic consoles. In the Caribbean, cost-effectiveness and health budgets are a constant discussion when it comes to technological advances in surgery. Some countries consider robotic surgery as a luxury more than a surgical need. Either way, surgeons and institutions aspiring to build robotic units should keep their populations’ needs in mind, the proportional necessities, and cost-effectiveness of their island, avoiding unsustainable technology disproportional to the island’s needs.

In developed countries, newer strategies to improve patient care have led to the implementation of ECMO programs and enhanced recovery after cardiac surgery (or ERACS) protocols in different institutions and centers^[19,20]^. Although these “newer” modern approaches to perioperative cardiac surgery patient care are still in their infancy even in developed mainland countries, low-income and developing countries are slowly establishing these protocols at their institutions. In the Caribbean, for example, the Cayman Islands and Martinique have the two most advanced ECMO programs with air-bridging for circulatory support in the Caribbean^[1,19]^. With regard to advancements in Caribbean CT surgery, Martinique and the Cayman Islands have the most advanced CT surgery departments^[1,19]^. Both perform minimally invasive cardiac and thoracic surgery, both have ECMO programs, the Cayman Islands have an LVAD program, and the cardiovascular surgery department of Martinique has been one of the firsts to research the European System for Cardiac Operative Risk Evaluation (or EuroSCORE) risk score^[1,19,21]^. In addition, Martinique will be introducing their congenital cardiac program by the end of 2020, as well as a robotic cardiac division by the end of 2021. Despite these achievements, Caribbean cardiac and thoracic surgery still have a long way to go, especially concerning governmental support and funding. Regardless of the middle to high-income profile of the Caribbean, there are significant differences in the speed of technological growth in CT surgery from island to island, as well as disparities between the quality of care and resources. Aruba, Martinique, and the Cayman Islands are good examples of islands that can easily afford to advance CT surgery when government policies, health budgets, and financial support strategies are implemented and established.

## CONCLUSION

The final destination of cardiac and thoracic surgery in the Caribbean depends not only on the support from local governmental policies and proper financial distribution of healthcare budgets, but efforts by the surgeons themselves to change and improve institutional cultures. Although resource availability still remains a challenge faced daily by local CT surgeons, the Caribbean remains an important region that deserves special attention with regard to the unmet needs and financial support for long-term sustainability. Ensuring quality and timely CT surgical care to an especially vulnerable population implies bringing first-class treatment closer to home.

**Table:** 

Authors' roles & responsibilities	
EEV	Substantial contributions to the conception or design of the work; or the acquisition, analysis, or interpretation of data for the work; final approval of the version to be published
TE	Substantial contributions to the conception or design of the work; or the acquisition, analysis, or interpretation of data for the work; final approval of the version to be published
RH	Drafting the work or revising it critically for important intellectual content; final approval of the version to be published
TFP	Drafting the work or revising it critically for important intellectual content; final approval of the version to be published
